# Development of a core outcome set for maternal and perinatal health research and surveillance in light of emerging and ongoing epidemic threats

**DOI:** 10.12688/gatesopenres.15136.2

**Published:** 2024-08-01

**Authors:** Veronica Pingray, Karen Klein, Juan Pedro Alonso, María Belizan, Gabriella Radice, Magdalena Babinska, Mabel Berrueta, Mercedes Bonet

**Affiliations:** 1 Department of Maternal and Child Health, Institute for Clinical Effectiveness and Health Policy, City of Buenos Aires, Buenos Aires, C1414CPV, Argentina; 2 Spanish and Portuguese Department, New York University, New York, New York, 10003, USA; 3 UNDP/UNFPA/UNICEF/WHO/World Bank Special Programme of Research, Development and Research Training in Human Reproduction (HRP), Department of Sexual and Reproductive Health and Research, World Health Organization, Geneva, Switzerland

**Keywords:** Pregnancy, newborn, epidemics, infectious, core outcome set, systematic review, Modified Delphi process

## Abstract

**Background:**

Maternal and perinatal health is often directly and indirectly affected during infectious disease epidemics. Yet, a lack of evidence on epidemics' impact on women and their offspring delays informed decision-making for healthcare providers, pregnant women, women in the post-pregnancy period and policy-makers. To rapidly generate evidence in these circumstances, we aim to develop a Core Outcome Set (COS) for maternal and perinatal health research and surveillance in light of emerging and ongoing epidemic threats.

**Methods:**

We will conduct a Systematic Review and a four-stage modified Delphi expert consensus. The systematic literature will aim to inform experts on outcomes reported in maternal and perinatal research and surveillance during previous epidemics. The expert consensus will involve two individual, anonymous online surveys to rate outcomes' importance and suggest new ones, one virtual meeting to discuss disagreements, and one in-person meeting to agree on the final COS, outcomes definitions and measurement methods. Four panels will be established to participate in the modified Delphi with expertise in (a) maternal and perinatal health, (b) neonatal health, (c) public health and emergency response, and (d) representation of civil society. We will recruit at least 20 international experts for each stakeholder group, with diverse backgrounds and gender, professional, and geographic balance. Only highly-rated outcomes (with at least 80% of ratings being 7–9 on a 9-point Likert scale) and no more than 10% of low ratings (1–3) will be included in the final COS.

**Conclusions:**

Implementing this COS in future maternal and perinatal research and surveillance, especially in the context of emerging and ongoing epidemic threats, will facilitate the rapid and systematic generation of evidence. It will also enhance the ability of policy-makers, healthcare providers, pregnant women and women in the post-pregnancy period and their families to make well-informed choices in challenging circumstances.

## Introduction

In recent decades, emerging and ongoing epidemic threats of infectious diseases have highlighted a range of direct and indirect health risks faced by pregnant women and their babies. Among these outbreaks were the 2009 H1N1 influenza pandemic ^1^; the Ebola epidemic in Guinea, Liberia, and Sierra Leone ^2^; the Zika virus outbreak in the Americas ^3^; and the COVID-19 pandemic ^4^. Outbreaks may increase the risk of severe morbidity or mortality in this special interest population. In addition, access to routine health services during outbreaks may be restricted, particularly in resource-limited settings ^5^. Given that these populations require ample and reliable healthcare services throughout the continuum of pregnancy and in the postnatal period, the risks of maternal and perinatal complications brought about by an outbreak are enhanced by the substantial restriction of access to quality care ^6, 7^.

The process of generating scientific evidence on how emerging and ongoing epidemic threats affect pregnant women and their offspring generally lags behind. This is further exacerbated by the fact that pregnancy status is often not reported in various diseases and other types of health surveillance systems (such as post-authorization safety or medical product utilization). Another factor is the exclusion of pregnant women from clinical trials of new or repurposed countermeasures (e.g., vaccines and drugs) ^8– 10^. As a result, decision makers, healthcare providers, and families of pregnant women lack the necessary scientific evidence to make informed decisions about maternal and perinatal health in the context of a disease outbreak ^10^.

Inequitable access to interventions to population-specific information regarding the burden and impact of disease, particularly in low- and middle-income countries (LMICs), highlights the need to strengthen and expand existing pregnancy research platforms in the context of epidemic and pandemic preparedness. Epidemic threats often advance rapidly, and the need for timely generation of knowledge and translations into policy and practice is critical. However, data are often times difficult to compare, summarize, and interpret across studies because of variations in the measured outcomes ^11^. These variations represent a substantial barrier to translating research into policy and public health interventions, and recommendations for clinical practice, all of which serve to protect the health of women and their babies during and after pregnancy in the context of emerging and ongoing infectious epidemic threats.

The identification of a set of core outcomes for measuring maternal and perinatal health during outbreaks, epidemics, or pandemics should facilitate generation and use of data in health-care decision making. A core outcome set has the potential to address inconsistencies in outcome selection, measurement, and reporting. Furthermore, it facilitates timely and efficient evidence generation ^12^. This outcome set should have the specificity to measure maternal and perinatal health metrics, and flexibility to be used in various study designs and in the context of various emerging and ongoing epidemic threats. Other COS have been developed related to maternal health and infectious diseases but do not respond to the needs this work is addressing. The Global Alignment of Immunization Safety Assessment in Pregnancy (GAIA) project ^13^ developed standardized case definitions for 21 perinatal outcomes to monitor immunization safety. However, these definitions are specific to the pharmacovigilance of vaccines.

### Objective

To develop a set of core outcomes on maternal and perinatal health to be reported in research and surveillance studies during emerging and ongoing epidemic threats, and to reach a consensus on the definitions of outcomes and methods of measurement, based on the results of a literature review and expert consensus-building process.

This protocol was developed following the COS-STAP (Core Outcome Set-STAndardised Protocol Items) Statement.

### Scope

***Health conditions and population.*** The domain of the study focuses on maternal and perinatal health. The COS will be applicable to epidemiological, clinical, and post-authorization research and surveillance studies concerning pregnant women, women in the postpartum or post-pregnancy period, and their offspring (embryos, fetuses, neonates) who are exposed to disease outbreaks (including epidemics and pandemics), in any country or setting. The study population includes all persons with gestational capacity, which here are being referred to as women.

An epidemic can be considered the simultaneous consolidation of multiple outbreaks over a wide geographical area. This usually involves the occurrence of a large number of new cases in a short time, which is greater than the expected number ^14^.

***Type of exposure.*** Any ongoing or emerging infectious disease epidemic, including those specified in the list of pathogens with epidemic and pandemic potential, regardless of the interventions eventually assessed. This may include, but is not limited to, Influenza, Ebola, Zika, Chikungunya, Dengue, Cholera, Yellow fever, and Coronaviruses of epidemic/pandemic potential.

***Context.*** The COS will be developed for use in maternal and perinatal epidemiological studies, clinical studies assessing the safety and efficacy of preventive and therapeutic interventions, and post-authorization safety, effectiveness, and disease surveillance in the context of disease outbreaks (including epidemics and pandemics).

It is expected that this COS will be applied in any country (including LMICs) or setting (community, primary health care, secondary, and tertiary care), in both rural and urban settings.

## Methods

### Study overview

We will define a set of maternal and perinatal outcomes to be reported in epidemiological studies, product development (vaccines and therapeutics), and post-authorization safety, effectiveness, and disease surveillance during emerging and ongoing epidemic threats following COMET guidelines ^12^, by conducting a systematic review of the literature and building consensus among key stakeholders.

### Systematic review to identify the list of outcomes

When developing a COS, the first step in determining “What to measure” is to explore existing knowledge about outcomes to inform the consensus process that follows. A systematic review of published and ongoing studies will be conducted according to the recommendations outlined in The Cochrane Handbook of Systematic Reviews of Interventions ^15^ and PRISMA ^16^. The purpose is to identify maternal and perinatal outcomes reported by researchers conducting studies during disease outbreaks, epidemics, or pandemics.

This systematic review will answer the following question: What are the maternal and perinatal health outcomes assessed during disease outbreaks, including pandemics and epidemics, in ongoing studies as well as those published in the period between 2015 and March 2023? The methods for this review are detailed in PROSPERO ( ID=CRD42023423196), and the search strategy is available at https://www.crd.york.ac.uk/PROSPEROFILES/423196_STRATEGY_20230502.pdf.

For data synthesis, the review team will systematically categorize each outcome under a new taxonomy for outcome classification that was developed from outcomes extracted from all published COS in the COMET database, selected Cochrane reviews, and clinical trial registries ^17^ to provide a structured conceptual framework and facilitate the presentation and interpretation of outcomes. The following categories may be considered as other options for group outcomes.

1. Mortality/survival (e.g., mortality/survival)
2. Physiological/clinical (e.g., physiological/clinical, general outcomes, gastrointestinal outcomes, infection and infestation outcomes, outcomes relating to neoplasms, musculoskeletal and connective tissue outcomes)
3. Life impact (e.g., functioning, physical, social, emotional well-being, global quality of life, cognitive, delivery of care)
4. Resource Use (e.g., resource use, economic, hospital, need for intervention, societal/care burden)
5. Adverse events/effects (e.g., adverse events/effects)

The outcomes will also be categorized according to the type of research: a) epidemiological studies to understand disease burden and severity, risk factors, and case ascertainment; b) intervention studies that aim to test novel or repurposed preventive and therapeutic interventions; and c) studies on post-authorization safety and effectiveness surveillance.

The outcomes identified through this systematic review will be considered in the Delphi survey.

### Consensus process

**Figure 1.**
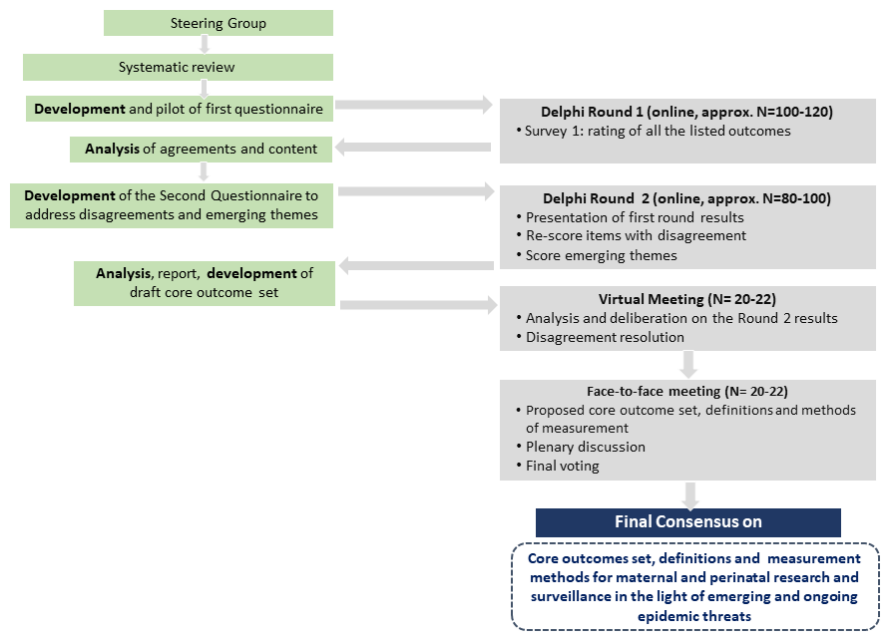
The iterative process planned to reach a consensus.

Expert consensus on a core outcome set for maternal and perinatal health to be reported in studies during emerging and ongoing epidemic threats will be achieved through an iterative fourth-stage modified Delphi process ( Figure 1). The consensus process will start with an international two-round online modified Delphi survey involving four key stakeholder panels; a virtual meeting to analyze and deliberate on the results of the second round, and resolve any disagreements; and conclude with a face-to-face meeting to finalize the above-mentioned COS and agree on the definitions of outcomes and methods of measurement. The Delphi process is a well-established tool with the advantage of being initially anonymous, thereby avoiding the overinfluence of dominant individuals and allowing participation from multiple geographies ^18^. The modified Delphi version was chosen, with a virtual meeting in the third round and a face-to-face meeting in the last round, because it enables a more effective interaction in the final round, given that further clarification or in-depth discussion might be required to resolve any potential disagreements ^19^.

Each expert panel should have between 7 and 15 members ^19^. We will recruit a minimum of 20 participants for each stakeholder group (maternal and perinatal health, neonatal health, public health and emergency response, and civil society), assuming a potential 20% attrition with a balanced gender and regional representation. Participant Information Sheets will be developed and piloted to clarify the terminology among representatives of the civil society.

### Online surveys

The first round will begin by administering a piloted questionnaire that will include the complete list of coded outcomes derived from the systematic review. Before starting the first survey, we will ask participants to verify their eligibility, interest in participating and commitment to participate in at least two surveys that will be conducted two months apart. Participants will be asked to rate the importance of each outcome to be reported in studies undertaken during disease outbreaks on a nine-point differential Likert scale ranging from 1 to 9 and categorized as:1–3 ‘not important’; 4–6 ‘important but not critical’; and 7–9 ‘critical.’ ^20^. Each questionnaire will have a set of closed-ended and open-ended questions to collect expert ratings, allow participants propose additional outcomes, and provide comments. Electronic surveys will be designed and administered by Delphi Manager ( www.comet-initiative.org/delphimanager/). Experts will be given three to four weeks to complete each survey.

The second survey will involve re-rating items which were the subject of disagreement within and among stakeholder groups. The additional outcomes listed by the participants will be incorporated into the second survey. To build a consensus, differing views need to be reconciled. Feedback between rounds will be presented to enable different opinions to be considered before re-rating an outcome.

Weekly reminders will be sent out during three consecutive weeks to participants who have not completed the survey with the intention of reaching a minimum of 80% response rate in each round. Participants will be asked to complete the surveys independently, anonymously, and in a manner that reflects their personal views. In round 1, they will also be asked to provide a short set of sociodemographic variables (i.e., age, gender, geographical location, and expertise).

### Online meeting

After completing the two online surveys, a purposive sample of approximately 20 experts representing four key stakeholder groups will review the findings from previous rounds and discuss all disagreements and draft the COS in an online meeting. Experts will be selected in a manner that respects equitable geographical representation of all six WHO regions, gender balance and diverse technical expertise.

### Face-to-face meeting to finalize the recommended COS

All participants of the online meeting will gather again at a WHO in-person consultation in Geneva, which will be dedicated to finalizing the above-mentioned COS and reaching a consensus on the definitions of the outcomes and methods of their measurement.

Presentations and discussions will be held in plenary sessions, and electronic platforms will be used to record the final decisions made anonymously by members of the expert group.

A guidance document will be offered to the participants prior to the consultation to help steer discussions and promote inclusiveness. The chair of the meeting will ensure that all participants can express their opinions freely and that their views are equally valued.

### Consensus definition

Following the COMET Handbook criteria ^12^ for retaining or dropping items between rounds, the following definitions will be applied to select core outcomes and determine agreement:

1. Agreement to retain an outcome: An outcome will be retained if rated from 7 to 9 (on a 9-point Likert scale) by at least 80% of all participants and from 1 to 3 by no more than 10% of the participants in each stakeholder group (i.e., each group has to agree that an outcome should be core for it to be included in the set).
2. Agreement to drop an outcome: An outcome will be dropped if rated 7 to 9 by no more than 50% of participants in each stakeholder group (i.e., there is no majority–more than 50% of participants in any group agrees that the outcome should be considered core).
3. No consensus: distributions differ from outcomes 1 and 2.

In cases where the systematic review yields an extensive list of outcomes and a greater-than-anticipated level of disagreement arises, we will apply more adaptable selection criteria, aligning them with the established criteria used by other research groups. These refined criteria will be clearly communicated to participants in the subsequent rounds of the process.

In addition, we will determine the degree of agreement stability by measuring the increase or reduction in variability of individual outcome ratings across rounds (comparison of interquartile ranges across rounds for each outcome).

### Stakeholders

To ensure an appropriate and acceptable core outcome set, it is essential to incorporate a diverse range of views and expertise. With this goal in mind, we will actively seek the participation of key stakeholders from around the world in the consensus process, to discuss, advise, and rate the core outcomes. We will divide the stakeholders into four distinct panels:

***Panel 1: Maternal and perinatal health.*** This panel will consist of senior clinical experts and researchers with proven knowledge and experience in the field of maternal and perinatal health. We are specifically looking for individuals who have expertise in antepartum, intrapartum, and postpartum care and research, as well as abortion and post-abortion care; preferably, who have conducted research or been involved in operational or policy aspects during previous epidemic threats. Midwives, nurses, obstetricians, gynecologists, anesthetists, general/family medical doctors, maternal critical care specialists, clinical investigators/trialists, and perinatal epidemiologists will be invited to participate in this panel. Regulators, funders, safety experts, and pharmacists working in the maternal and perinatal fields will also be invited.

***Panel 2: Neonatal health.*** This panel consists of senior clinical experts and researchers with proven knowledge and experience in the field of neonatal health (newborns up to 28 days of life). We are seeking individuals who have expertise in various aspects of neonatal care, including postnatal care, newborn immunization, breastfeeding, managing small and sick newborns, newborns with congenital abnormalities, and requiring admission to intensive care units. Preferably, panelists will have conducted research during previous epidemic threats. Nurses, neonatologists, pediatric infectious disease specialists, lactation consultants, and specialists in birth defects who meet the specified inclusion criteria will be invited to participate. Regulators, funders, safety experts, and pharmacists working in the neonatal and pediatric fields will also be invited.

***Panel 3: Public health and emergency response.*** This panel will consist of senior experts with proven knowledge and experience in planning (prevention and preparedness), management (mitigation, early response, recovery, and reconstruction), and responding to public health emergencies during epidemic threats at different levels (such as regional, national, and district). A range of health professionals with diverse backgrounds, including program managers, medical doctors, nurses, laboratory technicians and professionals, infectious disease experts, experts in public health, infection prevention and control experts, psychologists, social workers, epidemiologists, surveillance experts, and funders, will be invited to participate. It is preferable that these experts possess some experience in the field of maternal and perinatal health.

***Panel 4: Representatives of the civil society.*** This panel will consist of individuals with diverse backgrounds and areas of expertise dedicated to ensuring the well-being of women and families during the various stages of pregnancy, childbirth, and post-pregnancy, especially during epidemic threats. This panel will include women representatives, community advocates, and patient representatives.

### Participants recruitment

Special emphasis will be placed on including participants from LMICs and from geographies where outbreaks are common and practice and make decisions in less resourced settings.

Participants in panels 1, 2, and 3 will be selected from a wide range of groups and organizations, including the World Health Organization (WHO) collaboration networks such as Guideline Development Groups. Experts from the WHO regional offices and leading researchers in the field will also be considered.

Partnerships, alliances, and other non-governmental organizations (NGOs) dedicated to women and children's health, such as the Partnership for Maternal, Newborn, and Child Health (PMNCH) ^21^ and the IBP Network ^22^, among others, will be contacted to identify potential panelists with diverse expertise and practical experience to participate in Panel 4.

### Patient and Public Involvement

Representatives of the civil society will be invited to participate in online surveys to rate the proposed outcomes and propose additional outcomes not identified in the systematic review. This group will take the form of an independent panel to reduce the influence of professionals ^23^ and will be given as much weight as the rest of the panels. In the third round, 25% of the participants who will meet virtually and face-to-face to discuss the final COS will be representatives of women and the community of patients. This group will participate in the development and execution of the dissemination plan.

### Analysis

***Outcome scoring/feedback.*** Participants will be asked to rate the importance of each outcome on a nine-point differential Likert scale ranging from 1= ‘not important’ to 9= ‘critically important’ to be reported on studies during emerging and ongoing epidemic threats ^20^.

Feedback between rounds will be presented to enable the consideration of different opinions before re-rating an outcome. At the end of each round, the results for each outcome will be summarized and presented with descriptive statistics and graphical representations (histograms) when appropriate. The median rating of each stakeholder group will be incorporated into the second survey. The analysis of open-ended questions with openly stated reasoning behind the given ratings will be included in the feedback.

Open-ended questions and group discussions will be analyzed using content analysis. The responses will be coded by one researcher and reviewed by another researcher. Matrices will then be developed to help interpret these findings. The analysis process will be facilitated using the qualitative data management software Atlas.ti 8.4. All comments and opinions provided by the panelists will be included in the findings, regardless of whether one or more panelists mentioned them.

***Outcome definitions.*** During the systematic review of the literature, outcomes will be extracted using verbatim wording, and definitions will be grouped under the same outcome name. We will prepare a report describing different definitions, data sources, and frequencies.

The COMET handbook recommends that the outcome definition be generated after identifying the outcome set to deepen the discussion on a limited set of outcomes, rather than working with the original list, which is usually quite extensive. If the selected outcomes already have validated definitions previously published by the WHO, CDC, or Brighton Collaboration, it may not be necessary to discuss definitions based on the results of the systematic review with experts participating in the meetings. Conversely, some outcomes that do not have validated, detailed definitions may be selected, and in such a case, these definitions could be discussed and voted on during the face-to-face meeting.

***Measurement instruments.*** An outcome measurement instrument is a means by which a particular outcome is assessed and quantified. These instruments can take various forms, such as single questions, questionnaires, performance-based tests, physical examinations, laboratory measurements, and imaging techniques ^24^.

For each outcome included in the COS, we will list and describe the definitions and measurement instruments/techniques reported by authors in the following sources of information: a) systematic review that we will conduct in phase 1; b) other maternal and newborn health published COS; c) GAIA ^13^; and d) COSMIN database ( https://database.cosmin.nl/). To describe the psychometric properties of the instruments, we will review the COSMIN database of systematic reviews of measures of outcomes in addition to the above sources. The collected information will be reported and disseminated among participants as pre-reading materials. During the in-person meeting, the results will be presented, a plenary discussion will be facilitated, and participants will be asked to rate definitions and measurement methods guided by pre-established criteria, such as feasibility, accuracy, acceptability and resources required.

### Prospective Registration

This study will be prospectively registered with the COMET. This systematic review was registered in the International Prospective Register of Systematic Reviews (PROSPERO) with ID CRD42023423196. We will follow the Preferred Reporting Items for Systematic Reviews and Meta-Analyses (PRISMA) statement ( http://www.prisma-statement.org/) to report the systematic review findings, and the Core Outcome Set–STAndards for Reporting (COS-STAR) Statement to report the final COS ^25^.

### Reporting guidelines

The Core Outcome Set-STAndard for Reporting (COS-STAR) ^25^ will be used to report the COS. These guidelines were developed as reporting guidelines for COS studies. The COS-STAR statement comprises an 18-item checklist that has been deemed essential for complete and transparent reporting in COS studies. The checklist items are centered on the introduction, methods, results, and discussion sections of manuscripts describing the development of a certain COS. While it was developed by a panel of developers, methodologists, journal editors, potential users, and patient representatives, a possible limitation of COS-STAR is that it was largely developed without input from representatives from middle- or lower-income countries. The guidelines can still be applied to studies carried out in these settings, but may require some adaptations to encompass the additional challenges of developing COS in these settings. Given the numerous ongoing COS studies, the COS-STAR Statement is poised to serve as a valuable tool for enhancing the reporting of COS studies, ultimately benefiting all users of COS.

### Missing data

As part of the data management strategy, we will have designated staff to monitor missing data in the databases, and we will send reminders to participants if their records exceed a 20% missing data threshold.

The frequency of missing responses will be quantified and reported with the results of each Delphi round. If the second round shows more than a 20% loss of follow-up responses, then the rating distribution of the first round will be compared between participants who completed both rounds and those who completed only one round to compare and eventually identify any potential attrition bias.

### Ethics

***Ethics approval/informed consent.*** The modified Delphi process does not usually require an ethics approval. Nevertheless, the protocol and its annexes were submitted to the Research Ethics Review Committee (Geneva) and the RESPIRE Research Ethics Committee (Argentina) to assess whether they required a full review. This Delphi was exempt from WHO Ethics Review Committee review. However, invited participants will be asked to provide written consent and declare any potential conflicts of interest before completing the first online Delphi survey.

Potential external contributors to the development of the core outcomes set will be asked to complete the standard WHO declaration of interests form and declare any potential academic, professional, financial, or other conflict before invitations to participate are finalized. WHO staff will assess the declaration of interests and determine if a conflict of interest exists, and the risk of conflict adversely affects the development of the core outcome set. WHO standard criteria for assessing the severity of a conflict of interest will be used. Decisions are made on a case-by-case basis, depending on the severity of the conflicts of interest. Where a conflict of interest is not considered significant enough to affect an individual’s ability to make objective judgements or reduce process credibility, a summary of all disclosed interests will be reported in the final publication. When a declared conflict of interest is deemed serious enough, the expert will be precluded from participating in the process.

All participant information will be securely stored in the systems with password-protected access. Participants will be assigned unique identification codes to ensure anonymity in online surveys. Voting during consensus meetings will be anonymous through an electronic platform. Participants' information will be used to prepare individualized reports and follow-up between rounds. However, only the selected researchers will have access to the participants’ identification data. Opinions and comments by participants will not be attributed to any participant in any publication or report. Experts participating in the face-to-face meeting will be informed that the list of experts will be catalogued on the annex of the publication.

### Dissemination

The goal of this project is to integrate this core outcome set into all future epidemiological, product development, and post-authorization surveillance research on maternal and perinatal health conducted during emerging and ongoing epidemic threats. We will employ various methods to disseminate the core outcome set and encourage its adoption. The findings of the systematic review and consensus process will be published in peer-reviewed journals, shared with various research and surveillance networks, presented at national and international conferences, and promoted through community organizations and social media platforms. We will provide an overview of the core outcome set for the CROWN and COMET initiatives. Additionally, we will leverage social media platforms. To maximize the implementation of the final set of core outcomes, we will share the results with professional associations, relevant university research departments, and developers of clinical guidelines. By doing so, we aim to facilitate the widespread adoption of the core outcome set in the field of maternal and perinatal health research.

## Study status

At present, the team is in the concluding phases of analyzing the results of the second survey and making preparations for both the online meeting and the face-to-face meeting.

## Discussion

To ensure the health and safety of pregnant women and their children, the development of a COS to measure maternal and perinatal health during pandemics is imperative. Historically, this special interest group has been overlooked in medical research, which has created a knowledge gap that has hindered informed decision-making for healthcare providers, policy makers, as well as pregnant women and their families. While existing literature focuses on standardizing outcomes for pregnancy and newborns, particularly for immunization safety assessment, there are currently no published COS specifically designed for maternal and perinatal health in epidemic contexts. The development of a COS and its broad implementation will contribute to bridging this knowledge gap allowing for a swift and systematic evidence collection in diverse epidemiological studies, clinical investigations of preventive and therapeutic interventions, and post-authorization safety surveillance. One limitation is that consensus on outcome definitions and measurement methods will be established during in-person meetings and not validated by a larger group. Nevertheless, this COS will facilitate the generation of robust scientific data to support informed decision-making, ultimately improving health outcomes for pregnant women and newborns during epidemic circumstances. 

## Data Availability

No data are associated with this article. Open Science Framework: Maternal and perinatal outcomes during disease outbreaks: A systematic review. PRISMA-P Checklist, https://doi.org/10.17605/OSF.IO/8MNK6. Data are available under the terms of the Creative Commons Zero "No rights reserved" data waiver (CC0 1.0 Public domain dedication).
