# Supplementary material for: Development of a core outcome set for maternal and perinatal health research and surveillance in light of emerging and ongoing epidemic threats
Source: Gates Open Res. 2024 Aug 1;8:24. Originally published 2024 Mar 8. [Version 2] doi: 10.12688/gatesopenres.15136.2 (PMC11339271; doi:10.12688/gatesopenres.15136.2)
Supplement: Peer review history

# Reviewer report: Reviewer response for version 2

**DOI:** 10.21956/gatesopenres.17666.r37628

**Published:** 2024-08-21

**Authors:** Ushma Mehta

**Affiliations:** 1 University of Cape Town, Rondebosch, Western Cape, South Africa

The authors' responses are accepted . The authors have touched briefly on the limitations of this approach in the discussion. The anonymisation process is noted.

Is the study design appropriate for the research question?

Yes

Is the rationale for, and objectives of, the study clearly described?

Yes

Are sufficient details of the methods provided to allow replication by others?

Yes

Are the datasets clearly presented in a useable and accessible format?

Not applicable

Reviewer Expertise:

Maternal and child health, pharmacovigilance, post-marketing surveillance

I confirm that I have read this submission and believe that I have an appropriate level of expertise to confirm that it is of an acceptable scientific standard.

# Reviewer report: Reviewer response for version 1

**DOI:** 10.21956/gatesopenres.16474.r36722

**Published:** 2024-06-27

**Authors:** Kumanan Wilson

**Affiliations:** 1 University of Ottawa, Ottawa, Ontario, Canada

Is the rationale for, and objectives of, the study clearly described?

 This is a well described study.  The background is detailed and informative.  The study focusses on a very specific question - core outcome for maternal and perinatal health during pandemics.  The study protocol clearly describes the health condition and population, exposure and context.

- Is the study design appropriate for the research question?

The study describes methods for a systematic review and concensus process.  The methods are detailed for the latter.  The methods for the systematic review are summarised and the reader is referred to  a published protocol.  

 It would be useful to have a more detailed summary of the systematic review methods in this paper - perhaps as an appendix.  This can include the subquestions and a summary of the search strategy.

 The methods for the Concensus approach are appropriate and detailed.

- Are sufficient details of the methods provided to allow replication by others?

As above, it would be helpful to have more details on the systematic review component - perhaps as a flow chart figure.  

  

- Are the datasets clearly presented in a useable and accessible format?

Not applicable, although to see the search strategy for the systematic review the reader needs to click through 2 links (one to the protocol and then to a link to the search strategy in the protocol).

 Additional comments:

 The discussion is brief.

 It would be useful to include potential strengths, limitations, potential applications and any existing research on this question in the discussion.

Is the study design appropriate for the research question?

Yes

Is the rationale for, and objectives of, the study clearly described?

Yes

Are sufficient details of the methods provided to allow replication by others?

Partly

Are the datasets clearly presented in a useable and accessible format?

Not applicable

Reviewer Expertise:

Public health

I confirm that I have read this submission and believe that I have an appropriate level of expertise to confirm that it is of an acceptable scientific standard.

# Response

**Published:** 2024-07-31

**Authors:** Karen Klein

**Reviewer´s comment 1**

 The study describes methods for a systematic review and consensus process.  The methods are detailed for the latter.  The methods for the systematic review are summarised and the reader is referred to a published protocol. 

 It would be useful to have a more detailed summary of the systematic review methods in this paper - perhaps as an appendix.  This can include the sub questions and a summary of the search strategy.

 As above, it would be helpful to have more details on the systematic review component - perhaps as a flow chart figure. 

 (…) to see the search strategy for the systematic review the reader needs to click through 2 links (one to the protocol and then to a link to the search strategy in the protocol).

**Response to comment 1**

 We thank the reviewer for this comment because it helps to clarify this issue.

 The methods are detailed in PROSPERO ID=CRD42023423196, and the search strategy is also detailed here: https://www.crd.york.ac.uk/PROSPEROFILES/423196_STRATEGY_20230502.pdf

 Further details on the methodology and results of this systematic review will be published soon. In fact, you can access the preprint here: https://www.researchsquare.com/article/rs-4607012/v1.

 However, in response to this comment, we will add the link to the search strategy in the main text to facilitate access (line 95).

**Reviewer´s comment 2**

 The discussion is brief.

 It would be useful to include potential strengths, limitations, potential applications and any existing research on this question in the discussion.

**Response to comment 2**

 Thank you for your comment. We appreciate your feedback and agree that expanding the discussion would enhance the comprehensiveness of our manuscript.

 Here is how the discussion turned out (lines 372-387). We have underlined the modifications:

 To ensure the health and safety of pregnant women and their children, the development of a COS to measure maternal and perinatal health during pandemics is imperative. Historically, this special interest group has been overlooked in medical research, which has created a knowledge gap that has hindered informed decision-making for healthcare providers, policy makers, as well as pregnant women and their families. While existing literature focuses on standardizing outcomes for pregnancy and newborns, particularly for immunization safety assessment, there are currently no published COS specifically designed for maternal and perinatal health in epidemic contexts. The development of a COS and its broad implementation will contribute to bridging this knowledge gap allowing for a swift and systematic evidence collection in diverse epidemiological studies, clinical investigations of preventive and therapeutic interventions, and post-authorization safety surveillance. One limitation is that consensus on outcome definitions and measurement methods will be established during in-person meetings and not validated by a larger group. Nevertheless, this COS will facilitate the generation of robust scientific data to support informed decision-making, ultimately improving health outcomes for pregnant women and newborns during epidemic circumstances.

# Reviewer report: Reviewer response for version 1

**DOI:** 10.21956/gatesopenres.16474.r36718

**Published:** 2024-06-27

**Authors:** Ushma Mehta

**Affiliations:** 1 University of Cape Town, Rondebosch, Western Cape, South Africa

This protocol for a Delphi study to identify a core outcome set for maternal and perinatal health research and surveillance is well constructed and clear. Some comments and suggestions: 

 1) How will the study ensure that these COS can be globally applied including in all rural communities in LMIC settings? Will the participant selection approach will be adequate to mitigate the risk that these COS will not be universally applicable? 

 2) Limitations of such an approach of harmonisation need to be discussed particularly in relation to signal detection, assessment of causal association, prioritisation of certain outcomes in settings where other outcomes may pose a greater public health risk.

 3) Wouldn't  a more tiered approach to outcome sets  (e.g. tier 1, 2,  3 outcomes based on maturity, priorities and capacity of the healthcare system) that takes into consideration regional priorities, diagnostic capacity and available resources so that some settings could be leveraged to build on the core outcomes where suitable allowing for a more rapid generation of evidence that a more non-specific set of core indicators could overlook/ not detect?  

 3) While one appreciates the need to prioritise the development of such outcomes for epidemic threats. the health system and maternal and child clinical care and surveillance are not designed to only provide care for infectious diseases arising during epidemics and outbreaks but also for other communicable and non-communicable diseases as well. Is there a potential risk that this initiative could prioritse access to care for diseases arising during outbreaks over other key conditions affecting mothers and newborns?

 4) To assess whether there is any skewing in the representativeness of the research included in the systematic review, I suggest that the systematic review also reflect the geographic spread of the studies included and excluded from the review. 

 5) Provide details of what the self-screening tool will include.

 6) Given the small numbers, how will you ensure that the participants are truly anonymsed? It should be easy to identify individuals based on these sociodemographic variables.

 7) Should funders that are members of the health products industry should be not be specifically excluded from these panels?

 8) Under Patient and Public Involvement the authors refer to "independent panel" - who is this panel independent from? 

 9) How will patients and the public panel members be identified?

Is the study design appropriate for the research question?

Yes

Is the rationale for, and objectives of, the study clearly described?

Yes

Are sufficient details of the methods provided to allow replication by others?

Yes

Are the datasets clearly presented in a useable and accessible format?

Not applicable

Reviewer Expertise:

Maternal and child health, pharmacovigilance, post-marketing surveillance

I confirm that I have read this submission and believe that I have an appropriate level of expertise to confirm that it is of an acceptable scientific standard, however I have significant reservations, as outlined above.

# Response

**Published:** 2024-07-31

**Authors:** Karen Klein

**Reviewer´s comment 1**

 How will the study ensure that these COS can be globally applied including in all rural communities in LMIC settings?

 Will the participant selection approach will be adequate to mitigate the risk that these COS will not be universally applicable?

**Response to comment 1**

 We thank the reviewer for this reflection and the questions.

 Selected participants will be from a wide array of countries and regions, with varying income levels, ensuring the relevance and applicability of the developed COS across diverse settings, including LMICs.

 During the expert meetings, we plan to review all included outcomes and their definitions, measurement instruments, reliability, validity, and feasibility of data collection and reporting, with a special focus on LMICs.

**Reviewer´s comment 2**

 Limitations of such an approach of harmonisation need to be discussed particularly in relation to signal detection, assessment of causal association, prioritisation of certain outcomes in settings where other outcomes may pose a greater public health risk

 Wouldn't a more tiered approach to outcome sets (e.g. tier 1, 2, 3 outcomes based on maturity, priorities and capacity of the healthcare system) that takes into consideration regional priorities, diagnostic capacity and available resources so that some settings could be leveraged to build on the core outcomes where suitable allowing for a more rapid generation of evidence that a more non-specific set of core indicators could overlook/ not detect? 

**Response to comment 2**

 Thank you for this comment and this insightful contribution.

 Firstly, I would like to clarify that this COS will be applied in research during outbreaks, epidemics and pandemics. We agree with your observation, which highlights the importance of a COS for research. Pregnant women and their offspring are often severely impacted by outbreaks, which can increase maternal and neonatal morbidity and mortality. A core set of prioritized maternal and neonatal outcomes, along with their definitions and methods for measurement and reporting, will facilitate comparison and interpretation of findings and enable timely, informed decisions about care and risks, particularly in contexts where other outcomes may pose significant public health risks. We have clarified this concept in the objective: “To develop a set of core outcomes on maternal and perinatal health to be reported in research and surveillance studies during emerging and ongoing epidemic threats.”

 Secondly, given the complexity and breadth of the topic, we aim to develop a core set of outcomes for research, as well as complementary outcomes that can be applied in specific settings (depending on resource availability), for particular disease outbreaks, or different types of studies (e.g., epidemiological, product development, or post-authorization surveillance).

**Reviewer´s comment 3**

 While one appreciates the need to prioritise the development of such outcomes for epidemic threats. the health system and maternal and child clinical care and surveillance are not designed to only provide care for infectious diseases arising during epidemics and outbreaks but also for other communicable and non-communicable diseases as well. Is there a potential risk that this initiative could prioritse access to care for diseases arising during outbreaks over other key conditions affecting mothers and newborns?

**Response to comment 3**

 We greatly appreciate your concern and the question. The COS is designed to take into account general maternal and perinatal outcomes that need special attention in epidemic contexts, in addition to other specific outcomes related to the particular disease outbreak. It is precisely designed to collect data in response to the research needs of mothers and neonates in health emergency contexts (clinical or observational research studies, not for health system organization).

 This is explained in the rationale of the COS in the Introduction section.

**Reviewer´s comment 4**

 To assess whether there is any skewing in the representativeness of the research included in the systematic review, I suggest that the systematic review also reflect the geographic spread of the studies included and excluded from the review.

**Response to comment 4**

 We thank the reviewer for this comment. We will not restrict any locations for the published articles in the systematic review, which will adhere to Cochrane guidelines. We have added the following sentence: “We will not restrict either location or languages in the search strategy” (line 93).

 In addition, the systematic review was conducted to identify maternal and perinatal health outcomes reported in research conducted during outbreaks, epidemics, and pandemics, to provide a preliminary list that will serve as a foundation. The systematic review is just one of the sources but will be complemented with outcomes proposed by experts from all panels, giving special relevance to the representatives of the civil society and the public health and emergency response panel. The publication of the entirety methodological details and findings of the systematic review, including specifics on included and excluded articles based on geographical location and other criteria, will be available soon. You can now access the preprint here: https://www.researchsquare.com/article/rs-4607012/v1

**Reviewer´s comment 5**

 Provide details of what the self-screening tool will include.

**Response to comment 5**

 Thank you very much for allowing us to clarify this. Ultimately, it was not a tool but a self-assessment of eligibility.

 Before confirming participation, all experts invited to complete the survey were asked to self-verify their eligibility, interest, and commitment to participate. They were required to read and self-assess if they met the detailed criteria (possession of the necessary expertise for the panel they were invited to), interest in participating, commitment to respond to both surveys, and fluency in English or ability to read in English and respond in Spanish or Portuguese). Only those who met the criteria should proceed with the survey; others should not.

 We have deleted the phrase *'complete a self-screening tool to'* from line 141 to avoid confusion, since a tool was not ultimately used.

**Reviewer´s comment 6**

 Given the small numbers, how will you ensure that the participants are truly anonymized? It should be easy to identify individuals based on these sociodemographic variables.

**Response to comment 6**

 Thank you for this comment. To ensure that the participants remain truly anonymized, several measures will be implemented:

1. Unique identification codes for the online surveys: each participant will be assigned a unique identification code, ensuring that individual identities are not directly linked to their responses.
2. Centralized data management: only study staff will have access to the identification information, and all forms and databases with identifying information will be stored in a password-protected computer.
3. Anonymous voting in consensus meetings: during consensus meetings, where 20 experts may need to vote again, the process will also be anonymous, facilitated through an electronic platform.
4. Diverse sociodemographic variables: given the diversity of the participants in terms of countries, roles, and specialties, identifying individuals based on sociodemographic variables will be challenging, further protecting their anonymity.

We added the following paragraph: “Participants will be assigned unique identification codes to ensure anonymity in online surveys. Voting during consensus meetings will be anonymous through an electronic platform.” (Lines 347-349)

**Reviewer´s comment 7**

 Should funders that are members of the health products industry should be not be specifically excluded from these panels?

**Response to comment 7**

 We thank the reviewer for this question. We decided to include funders because this COS is expected to be applicable not only to epidemiological studies but also to product development and post-authorization surveillance during epidemics in the context of research. Therefore, the perspective of the funders is also considered relevant.

**Reviewer´s comment 8**

 Under Patient and Public Involvement the authors refer to "independent panel" - who is this panel independent from?

**Response to comment 8**

 Thank you very much for this question. That means that the representative of the civil society panel will be allowed to act independently from the other professionals. They will have the opportunity to meet and discuss separately, and make decisions without influence from maternal, neonatal, and public health experts.

**Reviewer´s comment 9**

 How will patients and the public panel members be identified?

**Response to comment 9**

 Thank you for the question. As explained in lines 243-246, partnerships, alliances, and other non-governmental organizations (NGOs) dedicated to women and children's health, such as the Partnership for Maternal, Newborn, and Child Health (PMNCH) and the IBP Network, among others, will be contacted to identify potential members from civil society with diverse expertise and practical experience to participate.
